# Impaired airway mucociliary function reduces antigen-specific IgA immune response to immunization with a claudin-4-targeting nasal vaccine in mice

**DOI:** 10.1038/s41598-018-21120-7

**Published:** 2018-02-13

**Authors:** Hidehiko Suzuki, Takahiro Nagatake, Ayaka Nasu, Huangwenxian Lan, Koji Ikegami, Mitsutoshi Setou, Yoko Hamazaki, Hiroshi Kiyono, Kiyohito Yagi, Masuo Kondoh, Jun Kunisawa

**Affiliations:** 1 Laboratory of Vaccine Materials, Center for Vaccine and Adjuvant Research and Laboratory of Gut Environmental System, National Institutes of Biomedical Innovation, Health and Nutrition (NIBIOHN), Ibaraki, Osaka, 567-0085 Japan; 2 Laboratory of Bio-Functional Molecular Chemistry, Graduate School of Pharmaceutical Sciences, Osaka University, Suita, Osaka, 565-0871 Japan; 3 International Mass Imaging Center and Department of Cellular and Molecular Anatomy, Hamamatsu University School of Medicine, Hamamatsu, Shizuoka, 431-3192 Japan; 4 Preeminent Medical Photonics Education & Research Center, Shizuoka, 431-3192 Japan; 5 Department of Anatomy, The university of Hong Kong, Hong Kong SAR, China; 6 Center for iPS Cell Research and Application (CiRA), Laboratory of Immunobiology, Graduate school of Medicine, Kyoto University, Kyoto, 606-8507 Japan; 7 Division of Mucosal Immunology, Department of Microbiology and Immunology and International Research and Development Center for Mucosal Vaccines, The Institute of Medical Sciences, The University of Tokyo, Tokyo, 108-8639 Japan; 8 Department of Immunology, Graduate School of Medicine, Chiba University, Chiba, 263-0022 Japan; 9 Graduate School of Pharmaceutical Sciences, Osaka University, Suita, Osaka, 565-0871 Japan; 10 Department of Microbiology and Infectious Diseases, Kobe University Graduate School of Medicine, Kobe, 650-0017 Japan; 11 Graduate School of Medicine, Graduate School of Pharmaceutical Sciences, and Graduate School of Dentistry, Osaka University, Osaka, 565-0871 Japan

## Abstract

Vaccine delivery is an essential element for the development of mucosal vaccine, but it remains to be investigated how physical barriers such as mucus and cilia affect vaccine delivery efficacy. Previously, we reported that C-terminal fragment of *Clostridium perfringens* enterotoxin (C-CPE) targeted claudin-4, which is expressed by the epithelium associated with nasopharynx-associated lymphoid tissue (NALT), and could be effective as a nasal vaccine delivery. Mice lacking tubulin tyrosine ligase-like family, member 1 (*Ttll1*-KO mice) showed mucus accumulation in nasal cavity due to the impaired motility of respiratory cilia. *Ttll1*-KO mice nasally immunized with C-CPE fused to pneumococcal surface protein A (PspA-C-CPE) showed reduced PspA-specific nasal IgA responses, impaired germinal center formation, and decreased germinal center B-cells and follicular helper T cells in the NALT. Although there was no change in the expression of claudin-4 in the NALT epithelium in *Ttll1*-KO mice, the epithelium was covered by a dense mucus that prevented the binding of PspA-C-CPE to NALT. However, administration of expectorant N-acetylcysteine removed the mucus and rescued the PspA-specific nasal IgA response. These results show that the accumulation of mucus caused by impaired respiratory cilia function is an interfering factor in the C-CPE-based claudin-4-targeting nasal vaccine.

## Introduction

Mucosal vaccines are used clinically to induce antigen-specific immune responses in mucosal tissue as the first line of defense against pathogens^1,2^. Secretory IgA is an effector molecule that prevents pathogenic invasion and neutralizes toxins^2^; therefore, mucosal vaccines must efficiently induce secretory IgA.

Mucosa-associated lymphoid tissues (MALTs) play a key role in the induction of antigen-specific secretory IgA responses in mucosal tissues. Nasopharynx-associated lymphoid tissue (NALT) is a representative MALT in the nose^3^. NALT has efferent, but not afferent, lymph as the conventional site of entry for antigens delivered by antigen-capturing dendritic cells. In addition, M cells exist at the NALT epithelium and act as antigen uptake cells from the nasal cavity to the NALT^3^. After processing, the antigens are presented to T cells and B cells located in specialized sites within the NALT called germinal centers (GCs), and B cells undergo IgA class switching with the help of follicular helper T cells (T_fh_ cells)^4^. Therefore, the delivery of antigens to the NALT is an important means of inducing antigen-specific secretory IgA responses.

The mucosal epithelium acts as a physical barrier to the uptake of antigen into MALT. This barrier function of epithelial cells is established by cell–cell connections called tight junctions, which are specialized connections between adjacent epithelial cells that hold the cells together and regulate the passage of materials across the epithelial membrane^5^. Tight junctions contain a variety of proteins, including claudins, occludin, tricellulin, angulins, and zonula occludens^5,6^. Although these tight junction molecules would at first appear to be preventive factors for vaccine delivery, they are actually prospective targets for the delivery of nasal vaccines. Indeed, we previously used *Clostridium perfringens* enterotoxin (CPE) to target the tight junctions in the mucosal epithelium associated with NALT. CPE binds to claudins in tight junctions through its C-terminus and forms a pore by polymerization through its N-terminus, which disrupts the barrier function of the epithelial layer and causes cytotoxicity^7^. Because claudin-4 is preferentially expressed in the mucosal epithelium associated with the NALT, including the M cells^8–10^, we used recombinant C-terminus of CPE (C-CPE) to deliver an antigen to the epithelium without inducing cytotoxicity^8,9^. In another study, we found that nasally administered pneumococcal surface protein A (PspA), a surface protein expressed by *Streptococcus pneumoniae*, fused to C-CPE (PspA-C-CPE) preferentially bound to NALT, including to M cells, and induced PspA-specific immune responses in the systemic and respiratory compartments^11^. We also confirmed that these immune responses were sufficient to protect against respiratory pneumococcal infection^11^.

In addition to the tight junction, mucus is another physical barrier to effective mucosal vaccination. Mucus is a slippery secretion composed of mucins, serum proteins, inorganic salts, and lipids suspended in water that is produced by goblet cells in the epithelium of the respiratory tract^12^. Disulfide bonds crosslink the secreted mucins to produce a viscoelastic gel that covers the epithelium and prevents attachment of exogenous materials. The amount of mucus on the surface of the respiratory epithelium is controlled by the beating of the mucocilia, which are hair-like, tubulin-based structures that project from the body of epithelial cells in the respiratory tract^13^. Several post-translational modifications of the tubulin subunits are necessary for the mucocilia to assume the correct curved morphology and to beat asymmetrically^14–17^. For example, tubulin glutamylation, which is catalyzed by tubulin tyrosine ligase-like protein 1 (Ttll1)^18^, adds several glutamic acids to the tubulin C-terminal tail domain, which is essential for ciliary function. We previously demonstrated that knockout of *Ttll1* in mice resulted in impaired tubulin glutamylation and a change in mucociliary morphology from the usual curved form to a straight form, which resulted in mucus accumulation in the nasal cavity due to a lack of asymmetry in the mucocilia beating cycle^14^.

In the present study, we used *Ttll1*-KO mice to evaluate how the mucocilia and mucus in the nasal cavity affect the antigen-specific immune responses induced by immunization with a C-CPE-based, claudin-4-targeting nasal vaccine.

## Results and Discussion

### Antigen-specific nasal IgA response was decreased in *Ttll1*-KO mice nasally immunized with PspA-C-CPE

**Figure 1:**
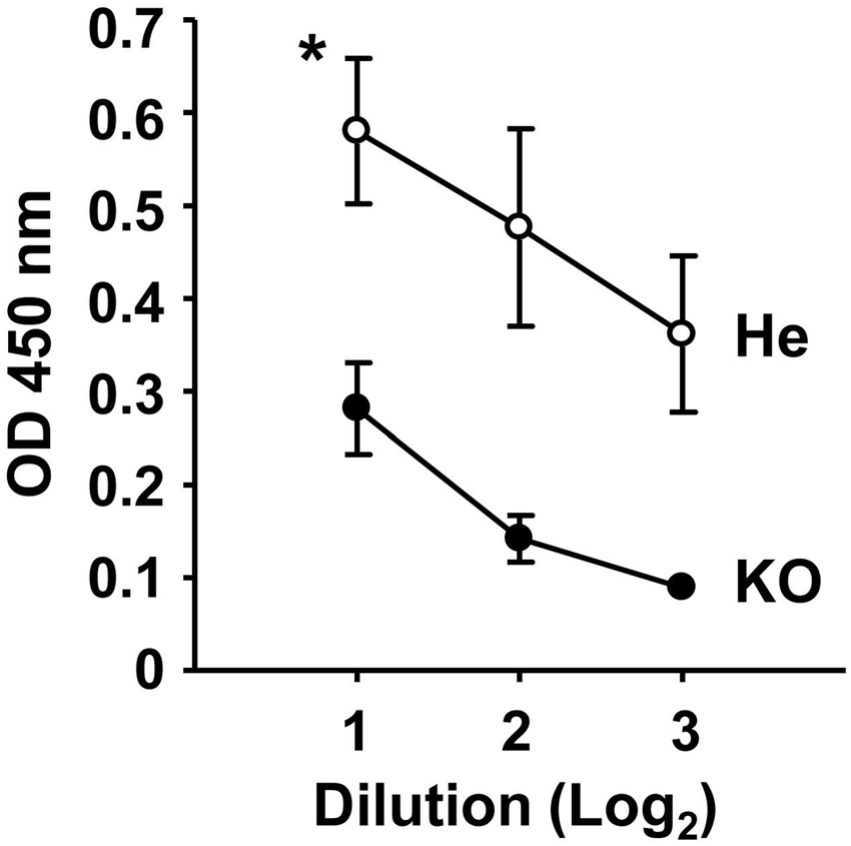
Antigen-specific nasal immune response was decreased in *Ttll1*-KO mice nasally immunized with PspA-C-CPE. *Ttll1*-hetero (He) and -knockout (KO) mice were nasally immunized with PspA-C-CPE once a week for three weeks. One week after the final immunization, PspA-specific nasal IgA was measured by means of an enzyme-linked immunosorbent assay. *Ttll1*-He mice, n = 4; *Ttll1*-KO mice, n = 3. Data are presented as mean ± SEM and are representative of two independent experiments. Values were compared by using Welch’s *t*-test. *P < 0.05. OD, optical density.

To examine whether airway mucociliary function affected the efficacy of the claudin-4-targeting nasal vaccine, we nasally immunized *Ttll1* mice with PspA-C-CPE once a week for three weeks. One week after the last immunization, we measured the concentration of PspA-specific antibodies with comparing *Ttll1*-hetero (He) to -KO mice. We first measured the concentration of PspA-specific antibodies in the nasal fluid. PspA-specific nasal IgA prevents colonization, or at least the initial stages of colonization, by *S. pneumoniae*^2^. The concentration of PspA-specific IgA antibody in the nasal fluid of *Ttll1*-KO mice was decreased compared with that of *Ttll1*-He mice (Fig. 1). In addition to PspA-specific nasal IgA, it is known that PspA-specific serum IgG eliminates *S. pneumoniae*^19^. Therefore, we also checked PspA-specific serum IgG. Unexpectedly, we found that the concentration of PspA-specific serum IgG was comparable between *Ttll1*-He and -KO mice (Supplementary Figure 1).

In addition to NALT, there are several alternative pathways through which immune responses can be induced. For instance, inducible bronchus-associated lymphoid tissue (iBALT) is induced by virus-based vaccine delivery (e.g., vaccinia virus vector), inflammation and infection^20–22^. The immunological structure and functions of iBALT are similar to those of other MALTs with regard to the initiation of antigen-specific immune responses^23–25^. Therefore, it is possible that nasal immunization with PspA-C-CPE induced the formation of iBALT as an inductive site for the systemic immune response in *Ttll1*-KO mice. Another possibility is the involvement of M cells in the respiratory epithelium^26^. The morphologic and immunologic functions of respiratory M cells, such as their short microvilli and the ability to take up vaccine antigens and pathogens (e.g., *Salmonella* spp.), are the same as those of the M cells in the NALT^26^. Thus, respiratory M cells appear to be an alternative pathway for the induction of systemic immune responses in *Ttll1*-KO mice.

We also checked the mice’s protective immunity against pneumococcal infection. Although PspA-specific nasal IgA was impaired in *Ttll1*-KO mice, survival rate was comparable between *Ttll1*-He and -KO mice (Supplementary Figure 2). In general, physical barriers such as mucus prevent the attachment of pathogens to epithelium. Therefore, it is likely that the dense nasal mucus in *Ttll1*-KO mice prevented the attachment of *S. pneumoniae* to epithelial cells and thus they showed low susceptibility to pneumococcal infection.

Together, these findings show that impaired airway mucociliary function prevented the induction of the nasal immune response.

### Immune responses in the GCs of NALT were impaired in *Ttll1*-KO mice immunized with PspA-C-CPE

Nasal vaccines are generally designed to deliver antigen to the NALT, which is the lymphoid tissue responsible for the induction of antigen-specific immune responses in the nasal tissues^3,27–29^. PspA-C-CPE also binds to NALT epithelium, and leads to the induction of PspA-specific immune responses^11^. To determine how the PspA-specific nasal IgA response was impaired in *Ttll1*-KO mice nasally immunized with PspA-C-CPE, we determined the frequencies and percentages of different types of cell in the NALT. Flow cytometric analysis revealed that the frequencies and percentages of B220^+^ B cells, CD11c^+^ dendritic cells, CD3^+^ T cells, CD3^+^CD4^+^ T cells, and CD3^+^CD8α^+^ T cells were comparable in the NALT of *Ttll1*-He and -KO mice (Supplementary Figure 3).

**Figure 2:**
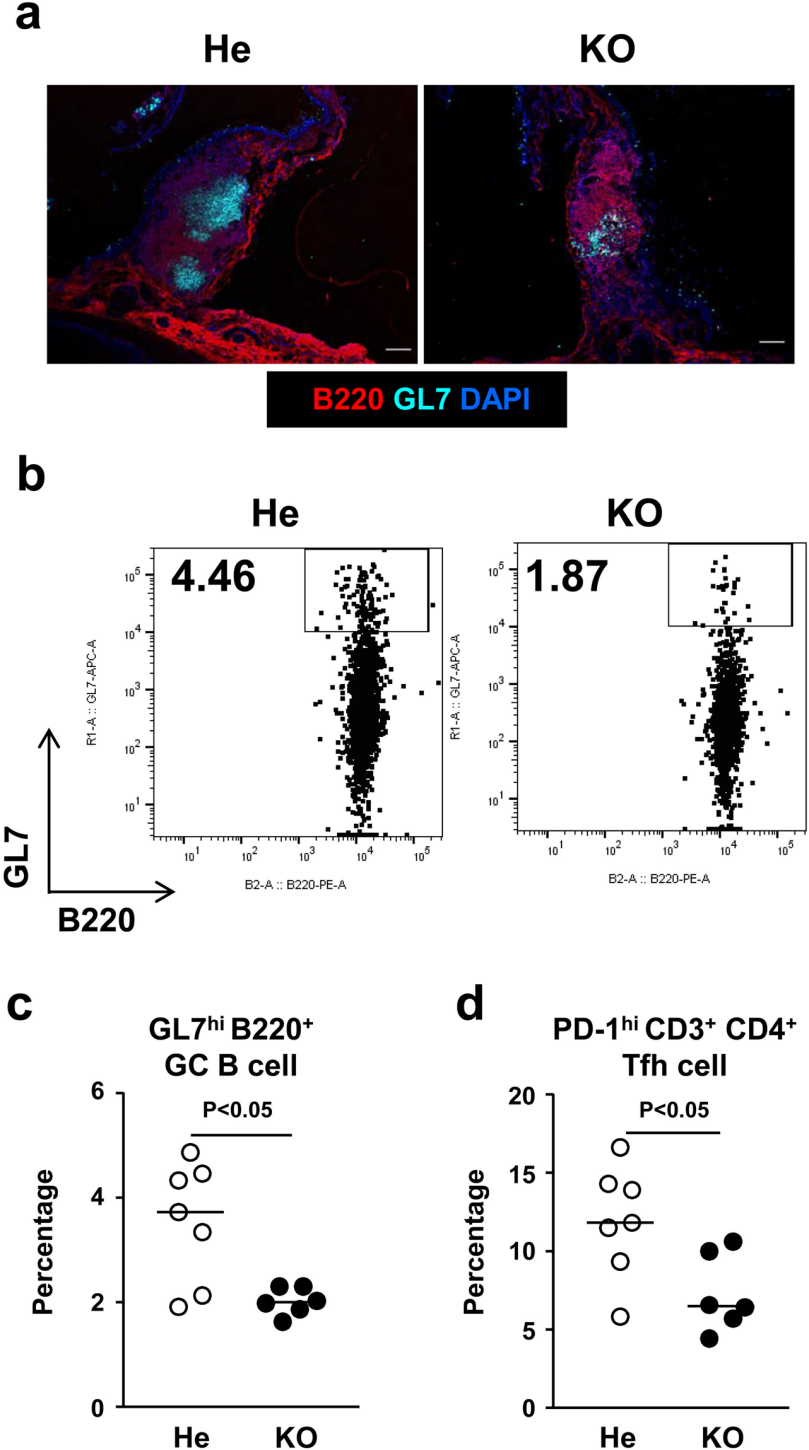
Immune responses in the germinal center of nasopharynx-associated lymphoid tissue were impaired in *Ttll1*-KO mice immunized with PspA-C-CPE. *Ttll1*-hetero (He) and -knockout (KO) mice were nasally immunized with PspA-C-CPE once a week for three weeks. (**a**) One week after the final immunization, sections of nasopharynx-associated lymphoid tissue (NALT) were stained with B220 (red), GL7 (light blue), and DAPI (blue). Scale bars, 100 µm. *Ttll1*-He, n = 5; *Ttll1*-KO, n = 4. (**b**–**d**). Frequency and numbers of germinal center (GC) B cells (**b**,**c**) and follicular helper T (T_fh_) cells (**d**) in the NALT were determined by means of flow cytometry. Bars indicate the median value. Data were collected from two separate experiments. Values were compared by using the non-parametric Mann–Whitney *U* test.

We next examined the cellular composition and formation of GCs in the NALT, where naïve B cells undergo IgA class switching upon antigen stimulation^30^. Nasal immunization with PspA-C-CPE induced GC formation and induced GL7^high^B220^+^ GC B cell proliferation in the NALT of *Ttll1*-He mice (Fig. 2a–c). However, GCs were smaller and had fewer B cells in the NALT of *Ttll1*-KO mice compared with *Ttll1*-He mice (Fig. 2a–c). Furthermore, the percentage of follicular helper T cells (T_fh_ cells), which play an important role in GC formation and IgA class switching^4^, was significantly lower in the NALT of *Ttll1*-KO mice compared with in the NALT of *Ttll1*-He mice (Fig. 2d).

These results show that impaired GC formation in the NALT was associated with the attenuation of the nasal IgA antibody response to nasal immunization with PspA-C-CPE in *Ttll1*-KO mice.

### Binding of PspA-C-CPE to the mucosal epithelium associated with the NALT was impaired in *Ttll1*-KO mice

We then examined the mechanisms underlying the impaired PspA-specific nasal IgA response that arose in *Ttll1*-KO mice nasally immunized with PspA-C-CPE. Immunofluorescence staining was used to examine the expression of claudin-4, the target molecule of C-CPE, in the mucosal epithelium associated with the NALT. Claudin-4 was highly expressed on the mucosal epithelium associated with the NALT in both *Ttll1*-He and -KO mice (Supplementary Figure 4), suggesting that the impaired antigen-specific nasal IgA response observed in the *Ttll1*-KO mice was not a result of reduced claudin-4 expression.

**Figure 3:**
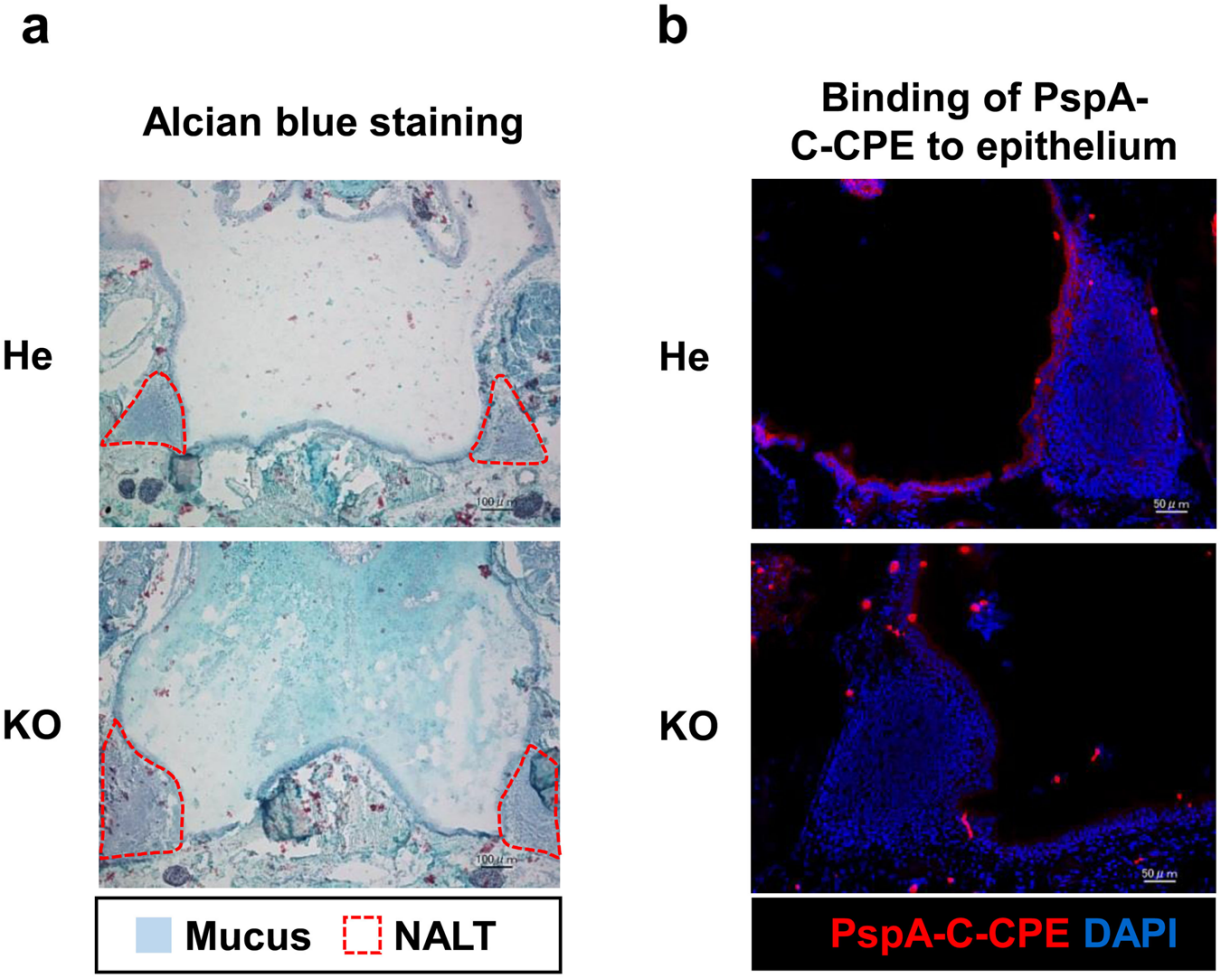
A dense mucus and reduced binding of PspA-C-CPE to the nasopharynx-associated lymphoid tissue epithelium was found in *Ttll1*-KO mice. (**a**) The mucus in sections of nasopharynx-associated lymphoid tissue (NALT) was stained with Alcian blue. *Ttll1*-He, n = 5; *Ttll1*-KO, n = 5. (**b**) *Ttll1*-hetero (He) or -knockout (KO) mice were nasally administered biotinylated PspA-C-CPE. Sections of NALT were stained with Alexa Fluor 546-conjugated streptavidin (red) and DAPI (blue). *Ttll1*-He, n = 3; *Ttll1*-KO, n = 3.

In a previous study, we found that impaired airway mucociliary motility caused mucus to accumulate in the nasal cavity of *Ttll1*-KO mice^14^, which led us to hypothesize that excessive amounts of mucus in the nasal cavity may prevent the binding of PspA-C-CPE to the mucosal epithelium associated with the NALT. Consistent with our previous findings^14^, in the present study we found that a dense mucus covered the NALT epithelium in *Ttll1*-KO mice but not in *Ttll1*-He mice (Fig. 3a). In addition, when we examined the intranasal distribution of PspA-C-CPE, we found that the binding of PspA-C-CPE to the mucosal epithelium associated with the NALT was attenuated in *Ttll1*-KO mice (Fig. 3b).

These findings indicate that accumulation of a dense mucus prevented the binding of PspA-C-CPE to the mucosal epithelium associated with the NALT, and therefore that the nasal vaccine was unable to induce PspA-specific nasal IgA response in *Ttll1*-KO mice.

### PspA-specific nasal immune IgA response was improved by removal of the nasal mucus in *Ttll1*-KO mice

**Figure 4:**
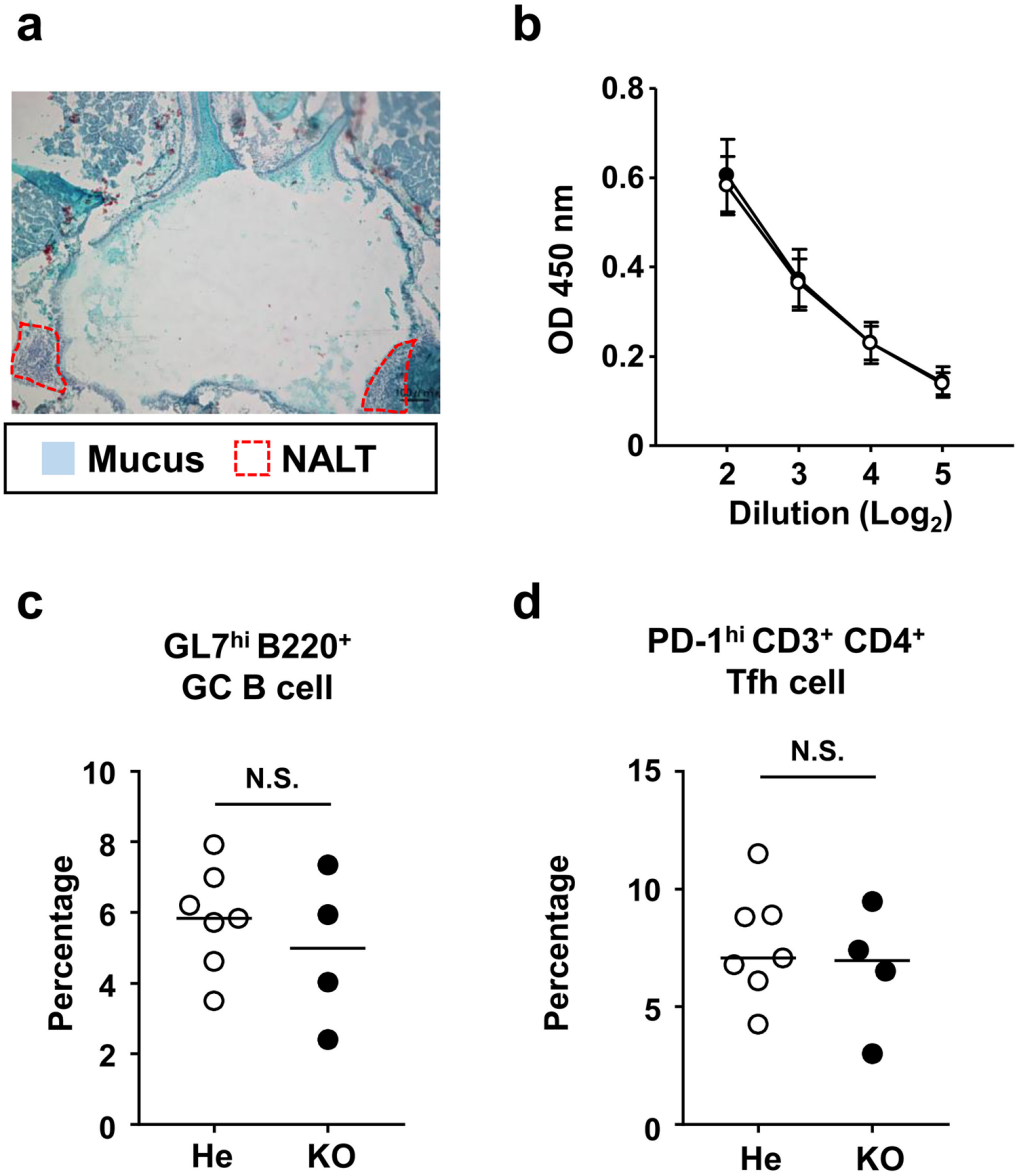
PspA-specific nasal immune IgA response was improved by removal of the nasal mucus in *Ttll1*-KO mice. (**a**) *Ttll1*-knockout (KO) mice were nasally administrated N-acetylcysteine. After 30 min, mucus in sections of nasopharynx-associated lymphoid tissue (NALT) was visualized by staining with Alcian blue. (**b**–**d**) Thirty minutes after N-acetylcysteine administration, *Ttll1*-hetero (He) (○) or -KO mice (●) were nasally immunized with PspA-C-CPE (once a week for three weeks). One week after the final immunization, the level of PspA-specific nasal IgA was measured by means of an enzyme-linked immunosorbent assay (**b**). Data are presented as mean ± SEM. Mononuclear cells were isolated from NALT and flow cytometric analysis was used to determine the percentages of germinal center (GC) B cells (**c**) and follicular helper T (T_fh_) cells (**d**). Bars indicate the median value. The data are representative of two independent experiments. Values were compared by using the non-parametric Mann–Whitney *U* test.

We hypothesized that the dense mucus covering the mucosal epithelium associated with the NALT in *Ttll1*-KO mice prevented the binding of PspA-C-CPE to the NALT epithelium, preventing the induction of the nasal IgA immune responses. Previous studies have demonstrated that removal of nasal mucus improves drug absorption in the nose^31^. We therefore removed the mucus by using N-acetylcysteine, which is a clinical expectorant that acts by cleaving the disulfide bonds between the mucin molecules in mucus^31^. We confirmed that the mucus was cleared from the NALT epithelium at 30 to 60 min after nasal administration of N-acetylcysteine in *Ttll1*-KO mice (Fig. 4a and Supplementary Figure 5). We also found that nasally administered PspA-C-CPE was retained at the mucosal epithelium associated with the NALT for 30 to 60 minutes after administration in C57BL/6 mice (Supplementary Figure 6). It is possible that reduced thiol could be reoxidized by air if longer extension time; therefore, based on these results, we nasally administered N-acetylcysteine to *Ttll1*-He or -KO mice followed 30 min later by nasal immunization with PspA-C-CPE. Our current findings suggested that *Ttll1*-KO mice without N-acetylcysteine treatment showed a decrease in PspA-specific nasal IgA together with impaired GC formation in the NALT because PspA-C-CPE was trapped by the dense nasal mucus. In contrast, the PspA-specific nasal IgA responses were comparable between *Ttll1*-He and -KO mice with N-acetylcysteine treatment (Fig. 4b). Furthermore, the percentages of GC B cells and T_fh_ cells were also comparable between *Ttll1*-He and -KO mice with N-acetylcysteine treatment (Fig. 4c,d). These results indicate that the dense mucus produced by the *Ttll1*-KO mice impaired the nasal immune responses induced by PspA-C-CPE, and that the removal of the mucus by administration of an expectorant rescued the impaired nasal immune response.

It is noteworthy that although the mucus was removed, the function of the mucocilia would have remained impaired, suggesting that the function of the mucocilia does not affect the efficacy of nasal vaccines. Allergies such as hay fever also cause mucus to accumulate in the nose. Therefore, in patients with allergies, removal of the nasal mucus either by using expectorants (e.g., N-acetylcysteine) or simply by blowing the nose immediately prior to immunization may ensure the complete induction of immune responses by nasal vaccines.

In summary, we elucidated the immunological role of airway mucociliary function with respect to delivery of a claudin-4-targeting nasal vaccine in *Ttll1*-KO mice, which possess straight rather than normal curved airway mucocilia due to impaired tubulin glutamylation, resulting in the loss of beating asymmetry and accumulation of a dense nasal mucus^14^. This dense nasal mucus prevented the binding of PspA-C-CPE to NALT epithelium, leading to reduced PspA-specific nasal IgA responses together with impaired GC formation in the NALT. Removal of the nasal mucus by using an expectorant rescued the nasal immune response. In addition to claudins, other tight junction proteins (e.g., occludin, tricellulin, angulins) are considered as targets for the delivery of nasal vaccines. For example, *Clostridium perfringens* iota-toxin binds to angulin-1, which is expressed by respiratory epithelium^32,33^. Since the present results indicate that vaccine delivery to NALT epithelium is affected by the accumulation of a dense nasal mucus, we conclude that nasal vaccines targeting occludin, tricellulin, and angulins may be possible but would similarly be affected by this accumulation of dense nasal mucus.

In this study, we used *Ttll1*-He mice as the controls for *Ttll1*-KO mice. We confirmed that the binding of PspA-C-CPE to NALT epithelium was identical between *Ttll1*-He and wild-type (WT) mice^11^. We also confirmed that *Ttll1*-WT and *Ttll1*-He mice showed comparable PspA-specific immune responses and GC formation in the NALT (Supplementary Figure 7a–d). In addition, we found that mucus removal had no effect on immune response induction in *Ttll1*-WT mice because *Ttll1*-WT mice did not show any accumulation of nasal mucus, which is consistent with the findings in *Ttll1*-He mice (Supplementary Figure 7e,f). These findings further suggest that mucus is a preventive factor for claudin-4-targeting nasal vaccine delivery.

Taken together, the present findings indicate that nasal mucus acts as a barrier against the delivery of nasal vaccines, and, therefore, that removal of nasal mucus is one approach to improve the efficacy of nasal vaccines.

## Methods

### Mice

*Ttll1*-KO mice (C57BL/6 background) were generated as previously described^14^. C57BL/6 mice were purchased from SLC, Inc. (Shizuoka, Japan). In the infection experiment, we killed the mice if their body weight was reduced by 20% or more. All experiments were approved by the Animal Care and Use Committee of the National Institutes of Biomedical Innovation, Health, and Nutrition (Approval Nos. DS27-47R1 and DS27-48R1) and were conducted in accordance with their guidelines.

### Preparation of the PspA-C-CPE fusion protein

pET16b plasmids encoding PspA or PspA-C-CPE were prepared as previously described^11^. To obtain recombinant protein, plasmids were transformed into *Escherichia coli* strain BL21 (DE3) (TOYOBO, Osaka, Japan). To induce the production of PspA or PspA-C-CPE, isopropyl-D-thiogalactopyranoside (Nacalai Tesque, Kyoto, Japan) was added to the culture medium. The culture pellet was sonicated in buffer A (10 mM Tris–HCl [pH 8.0], 400 mM NaCl_2_, 5 mM MgCl_2_, 0.1 mM phenylmethylsulfonyl fluoride, 1 mM 2-mercaptoethanol, and 10% glycerol). The supernatant was loaded onto a HiTrap HP column (GE Healthcare, Pittsburgh, Pennsylvania, USA). PspA or PspA-C-CPE protein was eluted with buffer A containing 100 to 500 mM imidazole. The solvent was exchanged with phosphate-buffered saline (PBS) by using a PD-10 column (GE Healthcare). The concentration of recombinant protein was measured by using a BCA Protein Assay Kit (Life Technologies, Carlsbad, California, USA). PspA-C-CPE was biotinylated by using a biotinylation kit (Thermo Fisher Scientific, Waltham, Massachusetts, USA).

### Immunization and mucus removal

Mice were nasally immunized with PspA-C-CPE (PspA: 5 µg, C-CPE: 2 µg) once a week for three weeks. One week after the final immunization, nasal fluid and serum were collected as previously reported^8^.

To remove nasal mucus, mice were nasally administered 15 µg of N-acetylcysteine (Sigma-Aldrich, St Louis, Missouri, USA). After 30 min, the mice were nasally immunized with PspA-C-CPE as described above.

### Enzyme-linked immunosorbent assay of PspA-specific production

The levels of PspA-specific IgA in nasal fluid and PspA-specific IgG in serum were measured by means of an enzyme-linked immune sorbent assay^11^. Ninety-six-well immunoassay plates were coated with PspA (0.05 µg/well) and incubated at 4 °C overnight. To prevent nonspecific binding, the plates were treated with 1% bovine serum albumin in PBS for 2 h at room temperature. After washing the plates with 0.05% Tween 20 in PBS, 2-fold serially diluted serum and nasal fluid were added to the wells and the plates were incubated for 2 h at room temperature. After washing the plates with 0.05% Tween 20 in PBS, goat anti-mouse IgA or IgG-conjugated with horseradish peroxidase (SouthernBiotech, Birmingham, Alabama, USA) was added to the wells and the plates were incubated for 1 h at room temperature. PspA-specific antibodies were detected by using 3,3′,5,5′-tetramethylbenzidine peroxide substrate. Optical density (wavelength 450 nm) was used an index of the progression of the color reaction.

### *S. pneumoniae* culture and infection

*S. pneumoniae* Xen10 (parental strain, A66.1 serotype 3; Caliper Life Sciences) were growth in brain–heart infusion broth at 37 °C under a 5% CO_2_ atmosphere with no aeration. *S. pneumoniae* Xen10 were washed and diluted with PBS. One week after the final immunization, mice were nasally challenged with 1.5 × 10^7^ CFU of *S. pneumoniae* Xen10. The survival of mice was monitored for 14 days.

### Cell isolation and flow cytometric analysis

To isolate mononuclear cells from NALT, NALT was first obtained from the upper jaw of the mice. NALT cells were isolated by gently rubbing the NALT sample with a needle under a stereoscopic microscope. After washing with PBS, the collected cells were treated with anti-mouse CD16/32 (clone 93; BioLegend, San Diego, California, USA) for 15 min at room temperature. After washing with PBS containing 2% newborn calf serum, the cells were stained with fluorescein isothiocyanate-conjugated hamster anti-mouse CD3ε (clone 145-2C11, BD Biosciences, San Diego, California, USA), phycoerythrin (PE)-conjugated rat anti-mouse B220 (clone RA3-6B2, BD Biosciences), PE-conjugated rat anti-mouse PD-1 (clone 29F1.A12, BioLegend), Alexa Fluor 647-conjugated rat anti-mouse GL7 (clone GL7, BioLegend), PE-Cy7-conjugated rat anti-mouse CD4 (clone RM4-5, BD Biosciences), PE-Cy7-conjugated Armenian hamster anti-mouse CD11c (clone N418, BioLegend), APC-Cy7-conjugated rat anti-mouse CD8α (clone 53-6.7, BD Biosciences), and Brilliant Violet 421-conjugated rat anti-mouse CD45 (clone 30-F11, BioLegend) for 30 min at 4 °C. After washing with PBS containing 2% newborn calf serum, cells were treated with 7-Amino-Actinomycin D (BioLegend) for 10 min at 4 °C and analyzed by means of flow cytometry (MACSQuant) (Miltenyi Biotec, Auburn, California, USA).

### Histochemical analysis of tissue specimens

To examine the expression of claudin-4 in NALT, NALT was embedded in Tissue-Tek optimal cutting temperature compound (Sakura Finetek Japan, Tokyo, Japan) and cut into 6-µm sections by using a cryostat. Sections were fixed in 100% acetone for 1 min at 4 °C. To prevent non-specific binding, sections were treated with 2% fetal calf serum in PBS for 30 min at room temperature. The sections were then washed with PBS and stained with anti-claudin-4 antibody^34^ at 4 °C overnight. After the sections were again washed with PBS, they were stained with Cy3-goat anti-rat IgG for 30 min at room temperature, washed again with PBS, and stained with 4′,6-diamidino-2-phenylindole (DAPI). After a final wash with PBS, the sections were mounted in Fluoromount (Diagnostic BioSystems, Pleasanton, California, USA) and observed by means of fluorescence microscopy (BZ-9000, Keyence, Osaka, Japan).

To stain mucus, skin and excess soft tissue was removed from the head of the mice, embedded in Super Cryo Embedding Medium (Section-lab, Hiroshima, Japan), and cut into 6-µm sections by using a cryostat. The sections were treated with 3% CH_3_COOH solution for 3 min at room temperature, stained with Alcian Blue Solution (Sigma-Aldrich), washed again with 3% CH_3_COOH solution, and stained with Nuclear Fast Red Solution (Sigma-Aldrich) for 1 min at room temperature. The sections were then washed with running water, mounted in Fluoromount (Diagnostic BioSystems), and observed by using an optical microscope.

To examine the binding of PspA-C-CPE to NALT epithelium, mice were nasally administered with biotinylated PspA-C-CPE (PspA: 5 µg, C-CPE: 2 µg). After 30 min, skin and excess soft tissue was removed from the head of the mice, embedded in Super Cryo Embedding Medium (Section-lab), and cut into 6-µm sections by using a cryostat. The sections were fixed with 100% acetone for 1 min at 4 °C, followed by treatment with 2% fetal calf serum in PBS for 30 min at room temperature to prevent non-specific binding. After washing with PBS, the sections were stained with Alexa Fluor 546-conjugated streptavidin and incubated at 4 °C overnight to detect biotinylated PspA-C-CPE, washed again with PBS, and stained with DAPI. The sections were then washed with PBS, mounted in Fluoromount (Diagnostic BioSystems), and observed by means of fluorescence microscopy (BZ-9000, Keyence).

### Data analysis

Data are presented as mean ± SEM. Statistical analyses were performed by using Welch’s *t*-test or the non-parametric Mann–Whitney *U* test (GraphPad Software, San Diego, California).

## Electronic supplementary material

Supplementary Figures
