# Supplementary material for: Impaired airway mucociliary function reduces antigen-specific IgA immune response to immunization with a claudin-4-targeting nasal vaccine in mice
Source: Sci Rep. 2018 Feb 13;8:2904. doi: 10.1038/s41598-018-21120-7 (PMC5811541; doi:10.1038/s41598-018-21120-7)
Supplement: Supplementary file 1 — Supplementary Figures [file 41598_2018_21120_MOESM1_ESM.pdf]

Supplementary Information

Impaired airway mucociliary function reduces antigen-specific IgA immune response to immunization with a claudin-4-targeting nasal vaccine in mice

Hidehiko Suzuki[1, 2], Takahiro Nagatake[1], Ayaka Nasu[1], Huangwenxian Lan[1], Koji Ikegami[3], Mitsutoshi Setou[3], Yoko Hamazaki[4], Hiroshi Kiyono[5,6], Kiyohito Yagi[2], Masuo Kondoh[2] and Jun Kunisawa[1,5,7,8*]

1 Laboratory of Vaccine Materials and Laboratory of Gut Environmental System, National Institutes of Biomedical Innovation, Health and Nutrition (NIBIOHN), Osaka 567-0085, Japan
2 Laboratory of Bio-Functional Molecular Chemistry, Graduate School of Pharmaceutical Sciences, Osaka University, Suita, Osaka 565-0871, Japan
3 Department of Cellular and Molecular Anatomy, and International Mass Imaging Center, Hamamatsu University School of Medicine, Hamamatsu, Shizuoka 431-3192, Japan
4 Department of Immunology and Cell Biology, Graduate School of Medicine, Kyoto University, Yoshida-Konoe, Sakyo-ku, Kyoto, 606-8501 Japan
5 Division of Mucosal Immunology, Department of Microbiology and Immunology and International Research and Development Center for Mucosal Vaccines, The Institute of Medical Sciences, The University of Tokyo, Tokyo 108-8639, Japan
6 Department of Immunology, Graduate School of Medicine, Chiba University, Chiba 263-0022, Japan
7 Department of Microbiology and Infectious Diseases, Kobe University Graduate School of Medicine, Kobe 650-0017, Japan
8 Graduate School of Medicine, Graduate School of Pharmaceutical Sciences, and Graduate School of Dentistry, Osaka University, Osaka 565-0871, Japan

*** Corresponding author.**
E-mail: kunisawa@nibiohn.go.jp (Jun Kunisawa)

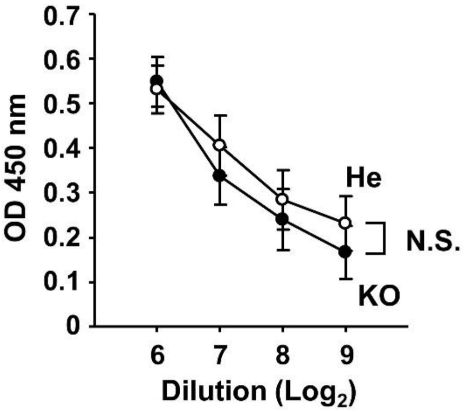

Supplementary Figure 1. Antigen-specific systemic immune response was not impaired in *Ttll1*-KO mice nasally immunized with PspA-C-CPE

Ttll1-hetero (He) and -knockout (KO) mice were nasally immunized with PspA-C-CPE once a week for three weeks. One week after the final immunization, PspA-specific serum IgG was measured by means of an enzyme-linked immunosorbent assay. *Ttll1*-He mice, n = 4; *Ttll1*-KO mice, n = 3. Data are presented as mean ± SEM and are representative of two independent experiments. Values were compared by using Welch's *t*-test. N.S., not significant.

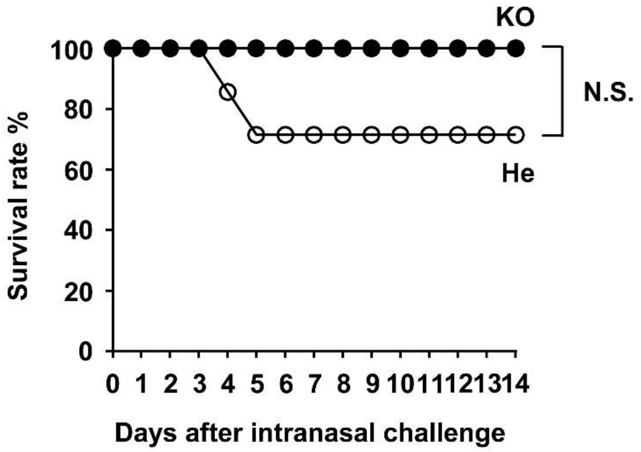

Supplementary Figure 2. Protective immunity against pneumococcal infection

Ttll1-hetero (He) and -knockout (KO) mice were nasally immunized with PspA-C-CPE once a week for three weeks. One week after the final immunization, mice were respiratory challenged with *S. pneumoniae* Xen10 (1.5×10^7 CFU/mouse), and their survival was monitored for 14 days. Survival rate was compared between groups by using the non-parametric Mann–Whitney *U* test. Data were collected from four separate experiments. *Ttll1*-He mice, n = 7; *Ttll1*-KO mice, n = 8. N.S., not significant.

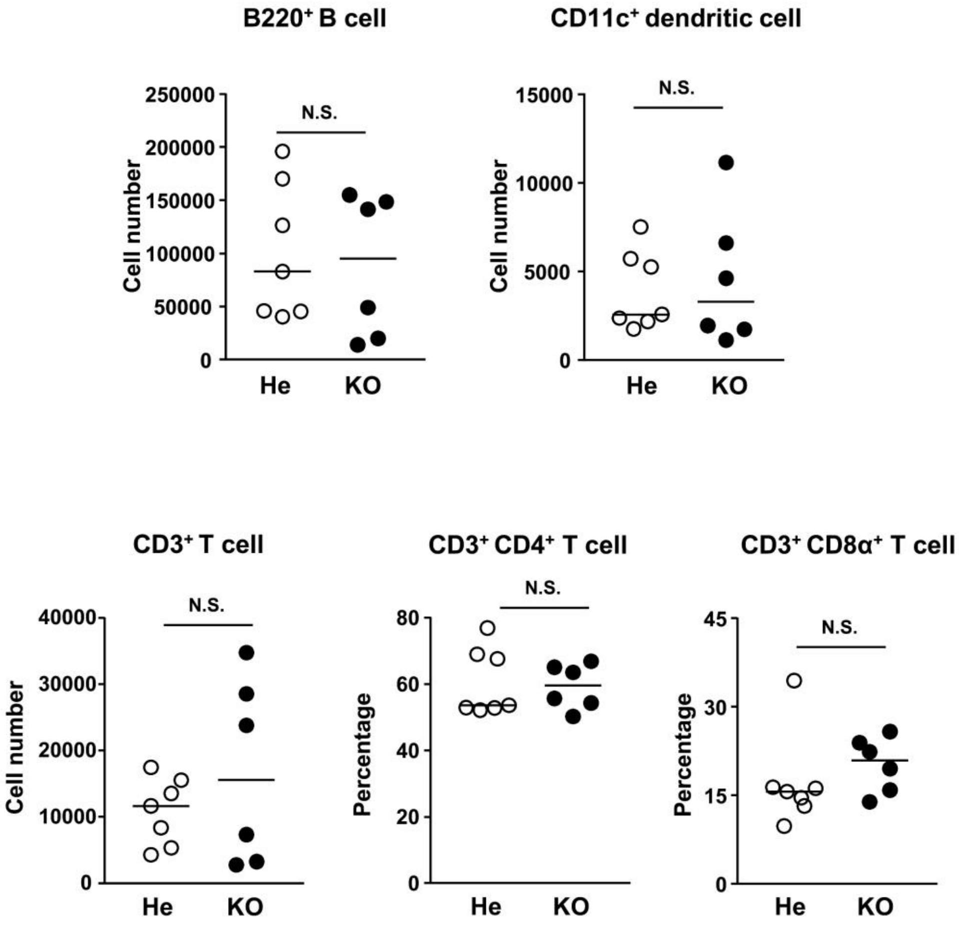

Supplementary Figure 3. Numbers of T, B, and dendritic cells were comparable in *Ttll1*-He and -KO mice

Ttll1-hetero (He) and -knockout (KO) mice were nasally immunized with PspA-C-CPE once a week for three weeks. One week after the final immunization, the frequency and cell numbers of B220$^+$ B cells, CD11c$^+$ dendritic cells, CD3$^+$ T cells, CD3$^+$CD4$^+$ T cells, and CD3$^+$CD8α$^+$ T cells in nasopharynx-associated lymphoid tissue were determined by means of flow cytometry. Bars indicate the median values. Data were collected from two individual experiments. Values were compared by using the non-parametric Mann–Whitney *U* test. N.S., not significant.

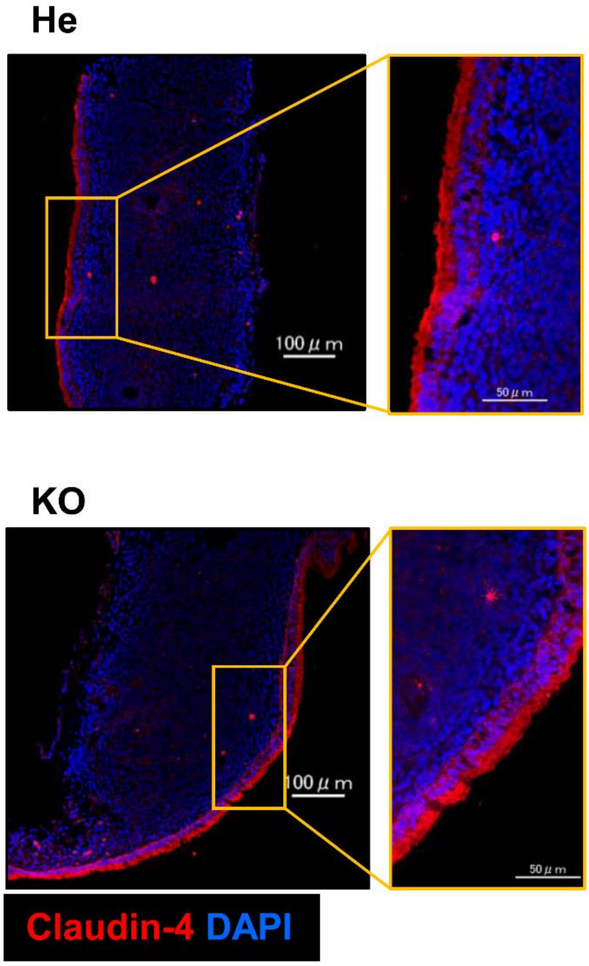

Supplementary Figure 4. *Ttll1*-KO mice showed normal expression of claudin-4 in mucosal epithelium associated with the nasopharynx-associated lymphoid tissue

Ttll1-hetero (He) and -knockout (KO) mice were nasally immunized with PspA-C-CPE once a week for three weeks. One week after the final immunization, sections of nasopharynx-associated lymphoid tissue were stained with anti-claudin-4 antibody (red). Nuclei were counterstained with DAPI (blue).

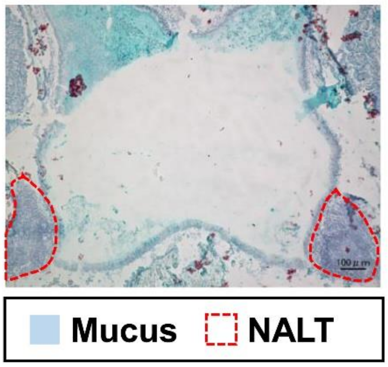

Supplementary Figure 5. Nasal administration of N-acetylcysteine removed the mucus from the mucosal epithelium associated with the nasopharynx-associated lymphoid tissue in *Ttll1*-KO mice

Ttll1-knockout (KO) mice were nasally administered N-acetylcysteine. After 60 min, the mucus in sections of nasopharynx-associated lymphoid tissue (NALT) was visualized by staining with Alcian blue.

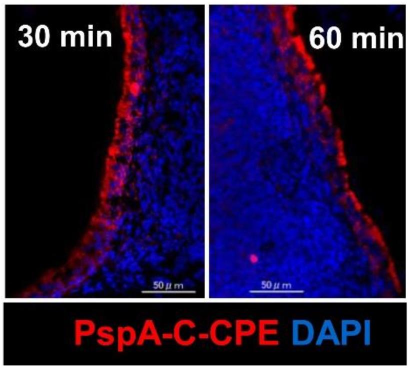

Supplementary Figure 6. Kinetics of PspA-C-CPE binding to the mucosal epithelium associated with the nasopharynx-associated lymphoid tissue in C57BL/6 mice

C57BL/6 mice were nasally administered biotinylated PspA-C-CPE. After 30 or 60 min, PspA-C-CPE binding to the mucosal epithelium associated with the nasopharynx-associated lymphoid tissue was visualized by staining with streptavidin (red). Nuclei were counterstained with DAPI (blue).

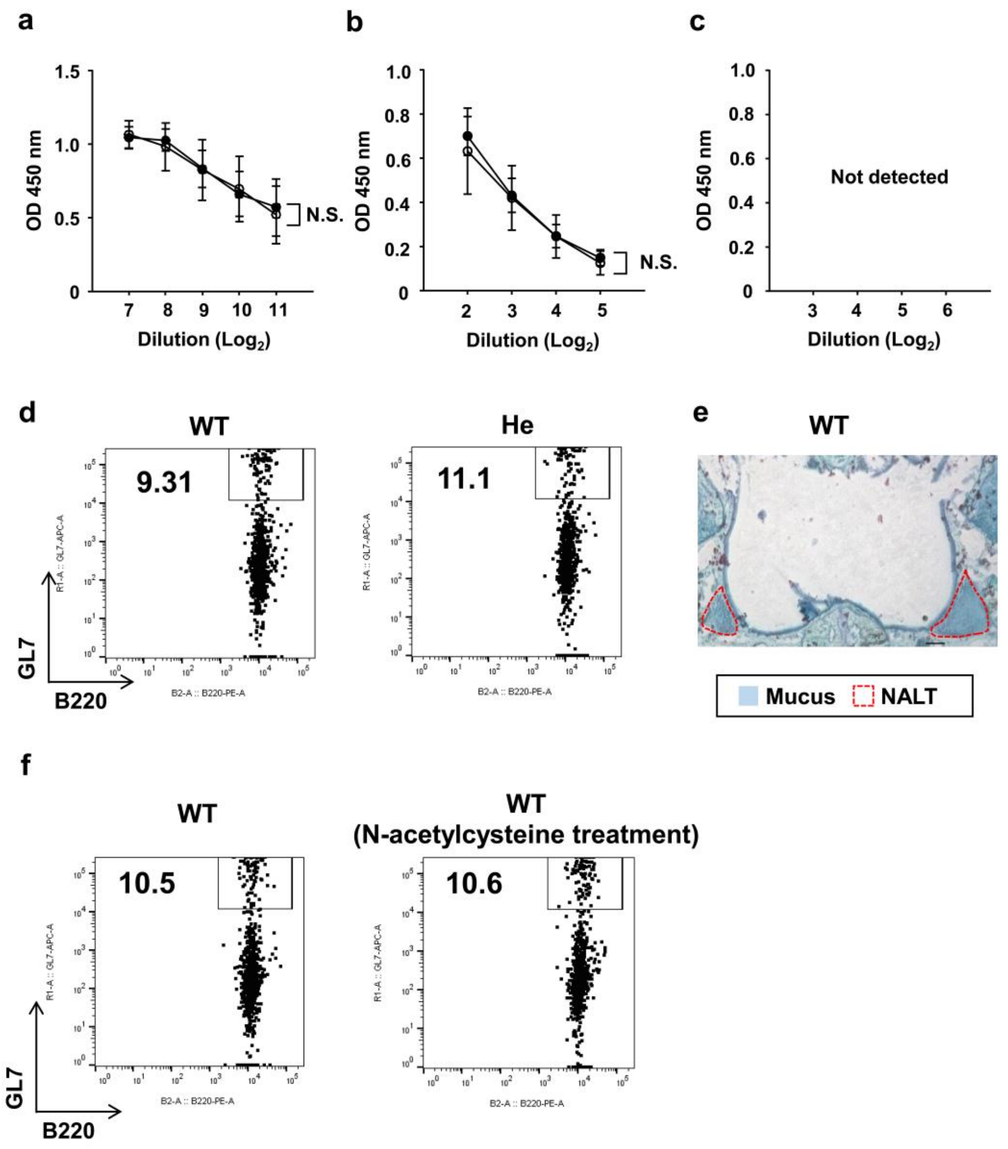

Supplementary Figure 7. Immune induction by PspA-C-CPE and accumulation of nasal mucus in *Ttll1*-WT and *Ttll1*-He mice

(a–c) *Ttll1*-wild-type (WT) (●) and *Ttll1*-hetero (He) mice (○) were nasally immunized with PspA-C-CPE once a week for three weeks. One week after the final immunization, PspA-specific serum IgG (a), nasal IgA (b), and nasal IgG (c) were measured by means of enzyme-linked immunosorbent assays. (d) Frequency of germinal center B cells in the nasopharynx-associated lymphoid tissue (NALT) was determined by means of flow cytometry. (e) Mucus in

sections of NALT was stained with Alcian blue. Scale bar, 100 μm. (f) *Ttll1*-WT mice were nasally administered N-acetylcysteine once a week for three weeks, and 30 minutes after N-acetylcysteine administration, the mice were nasally immunized with PspA-C-CPE. One week after the final immunization, the frequency of germinal center B cells in the NALT was determined by means of flow cytometry. Data are presented as mean ± SEM. Values were compared by using Welch’s *t*-test. OD, optical density. N.S., not significant.
